# Modified therapy concepts for fragility fractures of the pelvis after additional MRI

**DOI:** 10.1371/journal.pone.0238773

**Published:** 2020-10-08

**Authors:** Isabel Graul, Ivan Marintschev, Carsten Hackenbroch, Hans-Georg Palm, Benedikt Friemert, Patricia Lang

**Affiliations:** 1 Department of Trauma-, Hand- and Reconstructive Surgery, University Hospital Jena, Jena, Germany; 2 Department of Radiology and Neuroradiology, Ulm Army Hospital, Ulm, Germany; 3 Department of Orthopaedic and Trauma Surgery, Universitätsklinikum Erlangen, Erlangen, Germany; 4 Department of Trauma Surgery and Orthopaedics, Reconstructive and Septic Surgery, Sports Traumatology, Ulm Army Hospital, Ulm, Germany; University Hospital Zurich, SWITZERLAND

## Abstract

**Background:**

Fractures of the pelvic ring in elderly patients have increased in frequency over time. These injuries are associated with a high morbidity and have a socio-economic impact. The diagnostic procedures and their influence of therapy decisions are still controversial.

**Methods:**

In a retrospective study, we investigate the value of additional MRI examination on therapy decision of fragility fractures of the pelvis. The evaluation of all patients with pelvic fractures without adequate trauma and with performed CT and MRI was conducted at three large German hospitals. The imaging procedure took place within a maximum interval of 4 weeks. After evaluation of the imaging, the resulting therapeutic consequences either based on CT alone or on CT and MRI were reviewed by experienced pelvic surgeons.

**Results:**

Of 754 patients with pelvic injuries, 67 (age 80 +/- 9.7 years, f: m 54:13) could be included. The detection of vertical fractures in CT (n = 40 unilateral, n = 11 bilateral) could be increased by the additional MRI (n = 44 unilateral, n = 23 bilateral). A horizontal fracture component was identified in CT in 9.0% (n = 6) vs. MRI in 25.4% (n = 17) of the cases. An anterior pelvic ring injury was detected in 71.6% (n = 44; 4x bilateral) in CT, in 80.6% in MRI (n = 50, 4 bilateral). Additive MRI imaging increased the decision rate for surgical therapy from 20.9% (n = 14) to 31.3% (n = 21).

**Conclusions:**

The results of this study further support the value of bone marrow edema detection by MRI diagnostics (or dual source CT which showed promising initial results) for the detection of pelvic ring fractures. For the first time, the study identifies an additional therapeutic consequence by an increased rate of surgical procedures.

## Introduction

Due to the demographic development of an ageing population, the number of pelvic fractures in the elderly is increasing [1–3]. Through improved diagnostic possibilities, so-called insufficiency fractures of the pelvic ring (Fragility Fractures of the Pelvis, FFP) are diagnosed in patients without adequate or memorable trauma. The Availability of different diagnostic decision algorithms and associated therapies are often a challenge for the treating physician.

Older patients usually present with a minor trauma that is not remembered or that often occurred weeks ago. The leading symptoms are lumbosacral pain and immobility, but not neurological deficits or concomitant injuries. This requires a certain amount of experience in clinical and radiological diagnostics in order to attach adequate importance to the unspecific clinical picture and to initiate further diagnostics. Conventional x-rays often show no pathological findings or only a fracture of the pubic bone. The sensitivity of X-rays for the detection of a posterior pelvic ring injury is only 15–35% [4]. Further imaging is necessary. CT as a widely available, cost-effective and quickly available imaging is in the foreground with a detection rate of 53–75% [5]. MRI is available with a higher identification rate of bone edema 98–100% [6, 7]. MRI is more cost-intensive, limited and time-delayed. Nüchtern et al. described in a patient collective (n = 60) a detection rate of the posterior pelvic ring fracture in CT of 71%. This was 99% in the MRI. In the CT examination the posterior pelvic ring fracture was overlooked in 17% (n = 8) [8]. Similar results were shown in the study by Henes et al. (n = 38) with a detection rate of sacral fractures in CT of 66% and in MRI of 99%. These studies included patients with trauma who had an anterior pelvic ring fracture in x-ray [9]. Insufficiency fractures, however, were not included. The study by Pulley et al. retrospectively investigated the frequency of pelvic fractures in patients older than 50 years and low-energy traumas. He found a frequency of 16.7% (n = 19/114). Pulley diagnosed the fractures in CT and an MRI was supplemented only in some cases [10]. Insufficiency fractures were not detected. As far as the detection of FFP is concerned, there is only one study with two-digit case numbers by Cabarrus et al. which directly compares MRI and CT. Insufficiency fractures of the sacrum were detected in MRI in 100% (n = 67) and in CT in 75% of the cases. In the area of the anterior pelvic ring he showed a detection rate of 100% in MRI and 65.5% in CT. As a result, he recommends MRI as the diagnostic standard [6]. However, a common consensus has not yet been reached.

A new option, dual-energy CT (using the so-called virtual non-calcium technique) is derived from this.

According to WHO, the measurement of bone mineral density (BMD) using Dual Energy X-ray Absorptiometry (DEXA) is the method of choice [11]. Quantitative computed tomography (QTC) is another option, but has lost importance due to radiation exposure. There are already studies that have dealt with the screening of osteoporosis by determining bone density using conventional CT examination ("opportunistic osteoporosis diagnosis") [12]. This involves ROI measurement of the Hounsfield Units (HU), within the vertebral spongiosa in a normally configured vertebral body, preferably lumbar. There are already initial results showing a statistically significant correlation between the T-score from the DEXA measurement and the bone density measurement using CT [12]. It is not yet clear whether the HUs recorded in L5, for example, correlate with the severity of the insufficiency fracture. In severely injured patients with adequate pelvic trauma, diagnosis is clearly defined by the S3 guideline polytrauma [13]. In contrast, the diagnostic procedure for insufficiency fractures has not yet been conclusively clarified: An equivalent priority of MRI and Dual Energy CT (DECT) is described [7, 14, 15].

The aim of our retrospective multicenter study is therefore to analyse the sequence of the diagnostics performed on patients with suspected FFP and to outline the consequences of additional MRI imaging. The primary objectives are on the one hand to determine whether the additional MRI examination leads to a change in the CT-based FFP classification according to Rommens and on the other hand whether the additional MRI examination entails a modification of the therapy (conservative vs. surgical or change in the surgical procedure).

The Hounsfield units in L 5 as an indicator of reduced bone density were recorded as a secondary target.

## Patients and methods

### Patients

Three German hospitals with specialized trauma care and membership in the Pelvic Injury Register of the German Trauma Society participated in the retrospective multicenter study.

**Fig 1:**
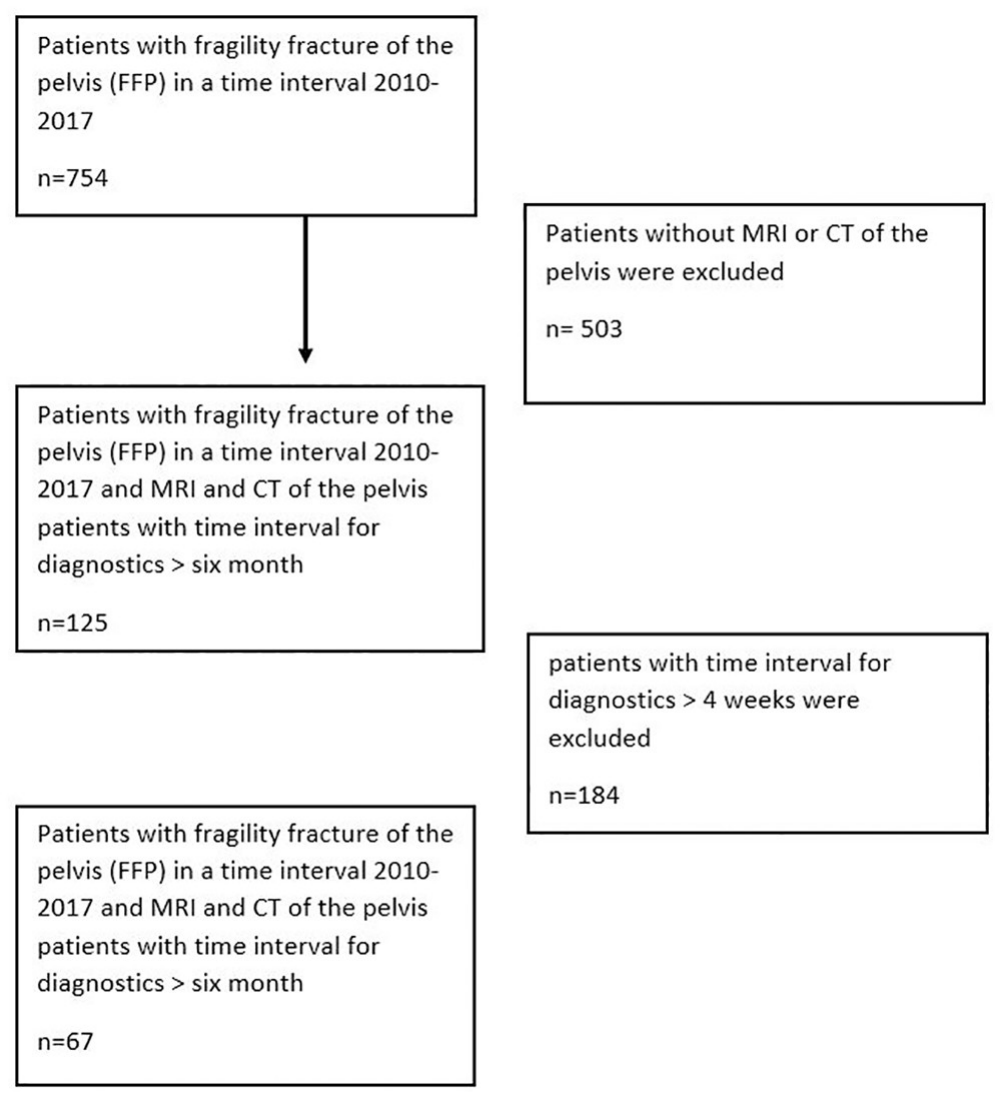
Schematic flow chart of the patient selection process.

All patients were included with unremarkable or inadequate trauma and resulting FFP and received both MRI and conventional CT with a maximum interval of 4 weeks to analyse fracture morphology in both imaging techniques from 2010 to 2017 (Fig 1). Since bone marrow edema changes persist for a very long time (over weeks) source, a period of 4 weeks was tolerated. All patients with FFP and incomplete diagnosis or extended period (>4 weeks) until diagnosis from CT and MRI or with adequate trauma anamnesis such as high energy injury were excluded.

Patients with FFP fractures were recorded for a period of seven years via internal clinical databases. In addition, all patients who did not meet the inclusion criteria were excluded. The fractures were classified in CT diagnostics according to the FFP classification of Rommens and Hofmann [16]. From this classification, a therapeutic regime can be derived that enables rapid and painless remobilization and reduces immobilization-associated risks (e.g. pneumonia, thromboembolism, skin ulcerations) presented in Table 1 [16].

**Table 1:** Overview of the fracture location and therapeutic regime recommended by Rommens et al. [16].

FFP type I	FFP type II	FFP type III	FFP type IV
anterior pelvic ring fracture	undislocated anterior and posterior pelvic fracture	dislocated anterior and posterior pelvic fracture	both sided anterior and posterior pelvic fracture
conservative	conservative try, if necessary operative	operative	operative
mobilization with pain-adapted full load, analgesia, osteoporosis therapy	conservative (see FFP I), in case of failure e.g. percutaneous screw osteosynthesis of the posterior pelvic ring	reduction and osteosynthesis of the anterior and posterior pelvic ring	reduction and osteosynthesis of the anterior and posterior pelvic ring

In the MRI diagnostics, the bone marrow edema was documented in regions defined by us (Ala, S1 vertebral body, S2 vertebral body, anterior pelvic ring). Furthermore, a measurement of the Hounsfield units was recorded in L 5 as an indicator of reduced bone density [12, 17]. Subsequently, the respective data sets were presented to three experienced pelvic surgeons (at least 10 years of professional experience with a focus on pelvic fractures) with the question whether and how they would classify the fracture according to FFP and how they would change the therapy decision by the additional information from the MRI examination. The change in therapy was defined as a change if at least to surgeons decided from conservative to surgical or in the type of surgical procedure.

In addition, patient-specific data such as "age", "sex", "underlying diseases", trauma history and "concomitant injuries" as well as inpatient treatment data such as length of stay and interval between CT and MRI are to be recorded and analysed. The parameters for each patient were listed using the Excel program (Microsoft Office 2016).

CT diagnosis was used as native CT of the pelvis in case of insufficiency fractures. Reconstruction was performed in the bone and soft tissue windows with sagittal and coronary reformations (multiplanar reconstructions (MPR)). Since each clinic uses different CT devices, there is no uniformity, the layer thickness was 1–5 mm. Different scanners were also used for MRI diagnostics, so that there is no uniform standard protocol. Edema expansion was assessed using fluid-sensitive fat-saturated sequences T2w or PDw sequences (Short-Tau Inversion Recovery (STIR)/ Turbo-Inversion Recovery-Magnitude (TIRM), Protone Densitiy weighted (PDw), Fat Saturation (fatsat)) and partially complementary T1w and T2w sequences.

In retrospective data evaluation, patients received an MRI if they complained of pain in the pelvic region, which could not be appropriately explained by CT imaging (no fracture detection and persistent pain). In patients who were seen to have a fracture in CT, MRI diagnosis was only performed if the fracture did not correspond to the symptoms. There are no internal clinical guidelines for the decision on MRI imaging. The procedure depends on the criteria mentioned above.

Statistical analysis was performed with SPSS V24.0 (IBM, Armonk, USA). Descriptive statistics were used for the basic variables. For continuous variables mean values and standard deviations (SD) were calculated. Fracture capture rates for CT and MRI were calculated using the previously described standard. Sensitivities were computed as a percentage of patients in whom at least one of the three techniques detected a fracture. To compare the groups, the two-sided student t-test for continuous variables and the Chi-square test and Fisher’s exact test for categorical variables were used. For the correlation of FFP classification to HU the Spearman-test was used. Results were considered statistically significant if at the corresponding p-value was < 0.05. The significance tests of CT and MRI sensitivities were calculated using the McNemar test. The study obtained approval by the Ethics Commission of the University of Jena (application number 2019–1380) and Ulm (application number 250/19) as well as registration in the German Register of Clinical Trials (DRKS; number DRKS00012440). Advice on data protection law has taken place.

## Results

Included were 67 patients (80+/-9.7 years, f:m 54:13) of the years 2010 to 2017 out of a total of 754 patients (88.9%). The time interval between CT and MRI of the pelvis was on average 4 days (range 1–21). The total hospital stay was 13 +/ -7.4 days. 31.3% (21/67) of the patients underwent surgical treatment. The hospital stay of the surgical group was significantly longer: 18 (+/-6.4) days vs. 10 (+/-6.4) days (p<0.01).

Dividing the underlying diseases into large groups with diseases of the blood vessels, heart, lungs, kidneys, musculoskeletal system (rheumatoid arthritis, chronic lumbar syndrome, etc.), malignancies, osteoporosis and endocrine diseases, resulted in 13% showing no relevant underlying diseases. On average, the patient suffered from 1.8 diseases (range 0–6) (Table 2). In 31.3% (21/67) of the patients no trauma anamnesis could be recorded, in 69.7% (46/67) a low-energy trauma usually consisted of a trip or fall from a sitting position. In 82.1% (55/67) the pelvic insufficiency fracture was a mono injury. Eleven patients had multiple injuries and showed 13 fractures, 1.2 fractures per patient (range 1–2) sorted by frequency: 6.0% (4/67) vertebral body fractures (1x L2, 1x L2 and L3, 1x T10, L1 and L2 and 1x L3), 4.5% (3/67) rib fractures, 3.0% (2/67) radius fractures, 3.0% (2/67) humeral fractures, 3.0% (2/67) acetabular fractures.

**Table 2:** Basic disease of the patients divided in vascular, cardial disease, diabetes, malignome, nephroplogical, neurological and pulmonal disease, osteoporosis, musculosceletal disease and no basic disease.

basic disease	vascular	cardial	diabetes	malignome	nephrological	neurological	pulmonal	osteoporosis	musculosceletal	none
amount of patients	38	20	6	11	5	14	8	14	9	9

Of the 67 patients, 4.5% (n = 3) showed no fracture, 19.4% (n = 13) FFP I, 50.7% (n = 34) FFP II, 9.0% (n = 6) FFP III, and 16.4% (n = 11) an FFP IV in CT and in MRI 7.5% (n = 5) an FFP I, 52.2% (n = 35) an FFP II, 3.0% (n = 2) an FFP III and 37.3% (n = 25) an FFP IV.

In the CT of the sacrum, 62 vertical fractures have been detected in 67 patients, of which 11 were bilateral fractures. The MRI of the sacrum revealed 90 vertical fractures, 23 of which were bilateral fractures. One transverse component was demarcated in CT in 9.0% (n = 6) vs. MRI in 25.4% (n = 17) of cases.

The anterior pelvic ring was fractured in CT 71.6% (n = 44; 4x bilateral), in MRI 80.6% (n = 50, 4x bilateral).

A fracture of the transverse process of L5 was shown in 9.0% (n = 6/67) in the radiological images.

In summary, 72.7% (n = 120/165) of the pelvic ring fracture components could be detected in the CT of the pelvis compared to the MRI of the pelvis, and all fractures could be detected in the MRI.

**Fig 2:**
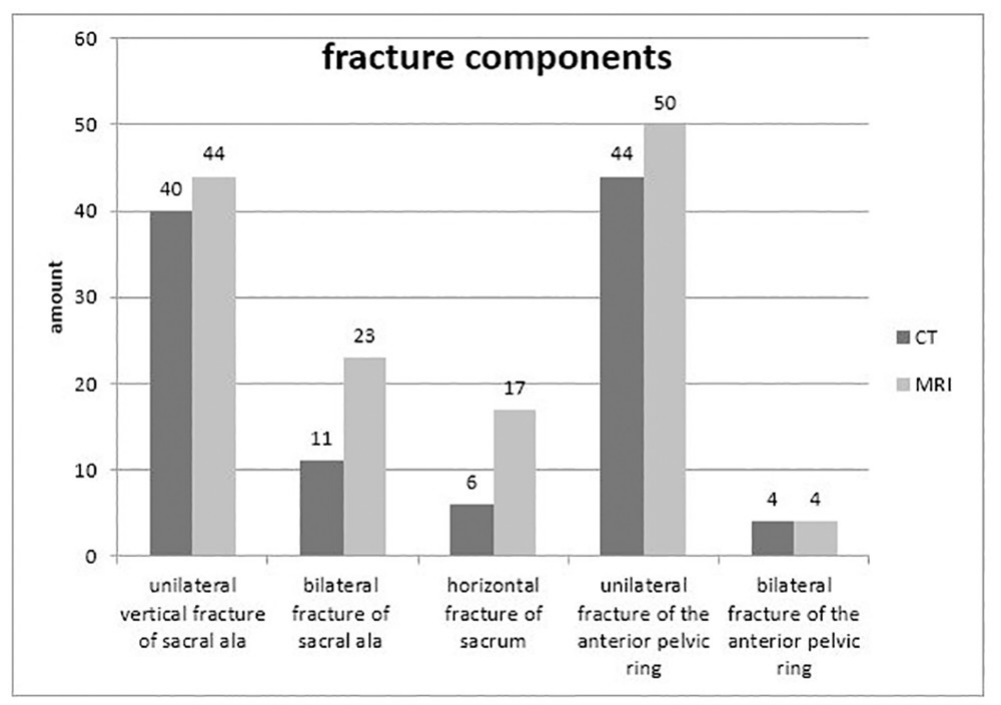
Distribution of the fracture location findings comparing CT with MRI: From left to right: Vertical unilateral fractures (40 vs. 44 patients), vertical bilateral fractures (11 vs. 23 patients), and horizontal fractures (6 vs. 17 patients), unilateral fracture of the anterior pelvic ring (44 vs. 50 patients) and bilateral fracture of the pelvic ring (4 vs. 4 patients).

19.4% (n = 13) of the patients with posterior pelvic ring fractures showed no simultaneous involvement of the anterior pelvic ring in CT or MRI. 36.4% of the sacral fractures showed an additional fracture component (vertical or horizontal fracture) in the MRI compared to the CT examination. (Fig 2).

Based on the classification of Rommens and Hofmann [16], the CT-based distribution shown in Table 3 was obtained.

**Table 3:** Distribution of all patients according to the fragility fracture of the pelvis classification according to Rommens comparing CT and MRI: No fracture in CT in 3 patients, FFP I (13 vs. 5 patients), FFP II (34 vs. 35 patients), FFP III (6 vs. 2 patients) and FFP IV (11 vs. 25 patients).

patient number	FFP in CT	therapy in CT	FFP in MRI	FFP change	therapy in CT and MRI	therapy change
1	0	conservative	II	yes	conservative	no
2	0	conservative	II	yes	conservative	no
3	0	conservative	IV	yes	operative	yes
4	I	conservative	I	no	conservative	no
5	I	conservative	I	no	conservative	no
6	I	conservative	I	no	conservative	no
7	I	conservative	I	no	conservative	no
8	I	conservative	I	no	conservative	no
9	I	conservative	II	yes	conservative	no
10	I	conservative	II	yes	conservative	no
11	I	conservative	II	yes	conservative	no
12	I	conservative	II	yes	conservative	no
13	I	conservative	III	yes	operative	yes
14	I	conservative	IV	yes	conservative	no
15	I	conservative	IV	yes	conservative	no
16	I	conservative	IV	yes	operative	yes
17	II	conservative	II	no	conservative	no
18	II	conservative	II	no	conservative	no
19	II	conservative	II	no	conservative	no
20	II	conservative	II	no	conservative	no
21	II	conservative	II	no	conservative	no
22	II	conservative	II	no	conservative	no
23	II	conservative	II	no	conservative	no
24	II	conservative	II	no	conservative	no
25	II	conservative	II	no	conservative	no
26	II	conservative	II	no	conservative	no
27	II	conservative	II	no	conservative	no
28	II	conservative	II	no	conservative	no
29	II	conservative	II	no	conservative	no
30	II	conservative	II	no	conservative	no
31	II	conservative	II	no	conservative	no
32	II	conservative	II	no	conservative	no
33	II	conservative	II	no	conservative	no
34	II	conservative	II	no	conservative	no
35	II	conservative	II	no	conservative	no
36	II	conservative	II	no	conservative	no
37	II	conservative	II	no	conservative	no
38	II	conservative	II	no	conservative	no
39	II	conservative	II	no	conservative	no
40	II	conservative	II	no	conservative	no
41	II	conservative	II	no	conservative	no
42	II	conservative	II	no	conservative	no
43	II	conservative	II	no	conservative	no
44	II	conservative	II	no	conservative	no
45	II	conservative	II	no	conservative	no
46	II	conservative	IV	yes	conservative	no
47	II	conservative	IV	yes	operative	yes
48	II	conservative	IV	yes	operative	yes
49	II	conservative	IV	yes	conservative	no
50	II	conservative	IV	yes	conservative	no
51	III	conservative	III	no	conservative	no
52	III	conservative	IV	yes	operative	yes
53	III	conservative	IV	yes	operative	yes
54	III	operative	IV	yes	operative	no
55	III	operative	IV	yes	operative	no
56	III	operative	IV	yes	operative	no
57	IV	operative	IV	no	operative	no
58	IV	operative	IV	no	operative	no
59	IV	operative	IV	no	operative	no
60	IV	operative	IV	no	operative	no
61	IV	operative	IV	no	operative	no
62	IV	operative	IV	no	operative	no
63	IV	operative	IV	no	operative	no
64	IV	operative	IV	no	operative	no
65	IV	operative	IV	no	operative	no
66	IV	operative	IV	no	operative	no
67	IV	operative	IV	no	operative	no

The therapy decision for isolated CT imaging and CT and MRI imaging is also presented.

The evaluation of the imaging diagnostics by an experienced pelvic surgeon without consideration of the clinical aspects was divided into the decision categories: Operative therapy with stabilization of the sacrum, operative therapy with lumbopelvic stabilization and conservative therapy.

Based on the isolated CT findings, 79.1% (n = 53/67) would have been treated with conservative therapy, 20.9% (n = 14/67) with surgical stabilization. Surgical stabilization (n = 14) was followed in 42.9% (n = 6/14) by isolated stabilization of the sacrum and in 57.1% (n = 8/14) by lumbopelvic stabilization.

Because of the combined CT and MRI findings 68.7% (n = 46/67) were subjected to conservative therapy, 31.3% (n = 21) to surgical stabilization. Surgical stabilization consisted of 42.9% (n = 9/21) sacral stabilization and 57.1% (n = 12/21) lumbopelvic stabilization.

Basing on additional MRI, changes in the treatment decision was evaluated in 10% (n = 7/67): In 6 cases, a change was made from conservative to surgical treatment and in one case from isolated stabilization of the sacrum to lumbopelvic stabilization. In the 6 cases with initial conservative treatment, there were two patients with FFP I in CT and four FFP II upgrading to FFP IV in MRI and following this, the operative way was aimed at. One case was an FFP III in CT upgrading to FFP IV in MRI and following this, the decision for lumbopelvic stabilization instead of isolated sacral stabilization was evaluated.

The Hounsfield units in L 5 were 62.7 +/- 33.7 HU. 10% of the patients showed values > 100 HU and therefore no clear bone density reduction in L 5.

**Fig 3:**
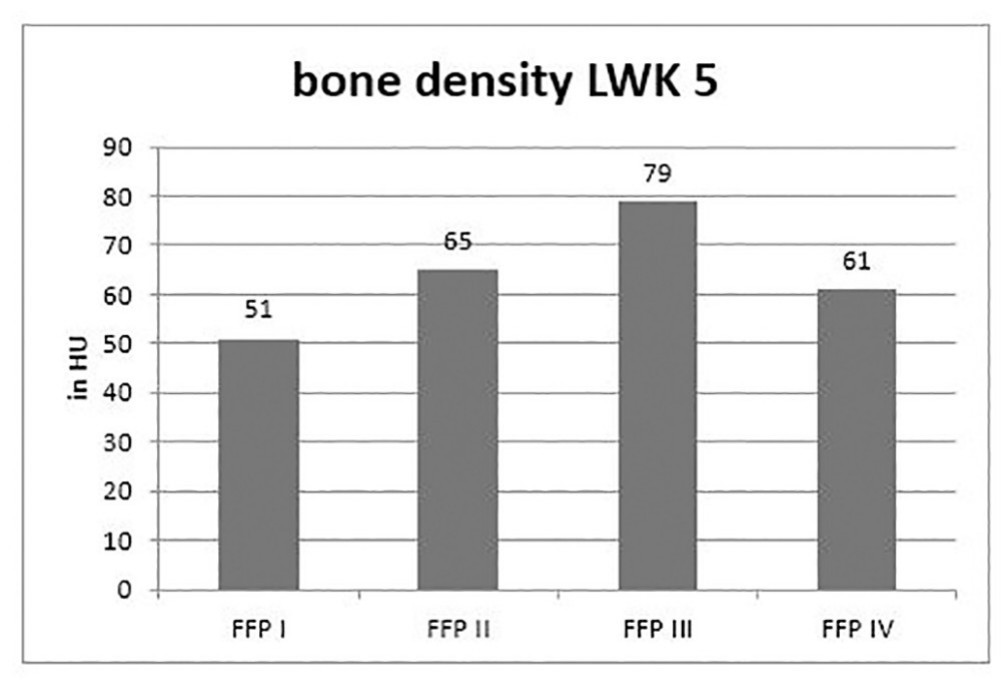
Distribution of patients bone mineral density in L5 according to the fragility fracture of the pelvis classification according to Rommens in CT.

A correlation of the Hounsfield units to the individual fracture classes FFP I-IV after Rommens could not be established. FFP IV fractures showed no lower bone density than FFP I, II or III. (Fig 3).

An anti-osteoporotic therapy was already taken at least as a basic therapy with calcium and vitamin D in 35.8% (n = 24/67). 64.2% (n = 43/67) did not receive anti-osteoporotic therapy.

## Discussion

The aim of the present study was to analyse the diagnostic procedure for 67 insufficiency fractures of the pelvis in a retrospective multicenter design and to derive a possible algorithm for diagnosis and therapy. Furthermore, a possible change of the FFP classification by the MRI diagnosis as well as a resulting change of the therapy should be recorded. The second aim was the correlation of bone density to fracture classification.

The second outcome parameter of the study, the correlation of the Hounsfield units in the vertebral body L5 as an indicator of bone density to the fracture classification could not show any correlation in the sense of a higher classification according to Rommens and Hofmann. Only 10% of the patients showed no clear bone density reduction based on the Hounsfield units in L 5. In 90% (n = 60/67) a reduced bone density could be measured in L5. Additive bone density measurements (DEXA or QCT) were not available. However, existing studies showed a correlation between reduced Hounsfield units and reduced bone density [8, 18]. An osteological diagnosis and possibly anti-osteoporotic therapy were indicated in surgical and conservative procedures.

The question of a quantitative regression of edema without fracture line in MRI with isolated anti-osteoporotic therapy cannot be answered on the basis of the data available.

Looking at the primary outcome parameter, fracture detection rates were statistically significant superior for MRI as compared to CT (100% MRI; 72.7% CT; p-value <0.05). The main reason for this is, the computed tomography is in terms of assessment of the posterior pelvic ring in the diagnosis of fragility fractures is limited and has its strengths in the diagnosis of the anterior pelvic ring. This led to an appreciation of the fracture classification to more severe injuries and thus to a more frequent decision on the surgical procedure. The surgical procedure was also partially influenced: Based on the therapy recommendations of Rommens et al. [19], lumbopelvic stabilization should be considered for additional transverse fracture components.

The present imaging diagnosis was examined for fracture signs. CT and MRI data were evaluated because of the FFP classification according to Rommens et al. [16].

In the present study, edema in MRI without adequate trauma was evaluated as fracture-associated bone bruise; this represents the histopathological correlate of microfractures of the cancellous bone with bleeding into the fatty bone marrow [20, 21]. In the absence of regeneration processes, the fracture becomes radiologically visible after 2–3 weeks in CT [22]. If the overload and the pathological bone metabolism can be eliminated, the development of a fracture can be avoided [23].

The question of the relevance of the additional MRI examination was assessed by investigating a change the therapy (conservative vs. surgical, or changing the surgical procedure) on the basis of findings from imaging diagnostics.

Patients with pain in a region of the pelvis without corresponding fracture detection in CT should be diagnosed by MRI or DECT. The indirect fracture signs of oedema with or without a delimitable fracture line are often visible here. Cabarrus et al. describes this in 7% of their patients [6]. With continued immobility and fracture signs visible in MRI, it remains controversial whether surgical intervention is indicated to avoid immobilization-associated complications [24, 25]. The question of a bilateral stabilization of sacrum fractures with only unilateral fractures in CT and additional contralateral edema in MRI to avoid subsequent operations by a contralateral follow-up fracture is also controversial.

It is undisputed that the decision on therapy in FFP is not only influenced by imaging, but also by mobilization and patient-specific factors such as dementia, age, concomitant diseases [25]. Nevertheless, imaging is an important influencing parameter for therapy decisions. This raises the question of whether a therapy recommendation can be made on the basis of the classification according to Rommens et al [16] In our study, 31% (21/67) patients showed a change in fracture classification, but only 10% (7/67) changed their treatment decision. The decisive factor here was therefore not the classification alone, but other factors. Since the clinical facts were not available, the localization of the additively determined edema was the main factor in the decision making process. Based on the therapy recommendations of Rommens et al. [16], lumbopelvine stabilization must be considered for additional transverse fracture components. Our data show that each FFP IV classified fracture resulted in a surgical therapy decision based only on image morphology. This was not the case in CT and MRI for the FFP IV entity classified in MRI. A decision for lumbopelvic stabilization was made to a small extent and only in the presence of a fracture line with dislocation. In our study a decision for lumbopelvic stabilization was not made only because of the edema on MRI. The FFP classification isolated cannot be used in MRI in our study to make a decision.

Due to the lack of agreement in dealing with oedema detected in MRI with or without fracture lines without correlation in CT, an additive MRI can only be recommended to a limited extent. Further studies on the value of the isolated oedema visible on MRI are necessary to establish uniform therapeutic consequences. A limitation of the study is the sample considered to be not representative of the underlying overall population in a clinical practice setting. Different devices were used, so that there was no uniform protocol for retrospective analysis.

The FFP classification is defined by the CT and the presence of edema in the MRI has not yet been taken into account. However, our interpretation of the classification in MRI is based on the proof of edema, so that it is necessary to critically question the disease value of the edema. However, we would like to encourage discussion as to whether it would not make sense to supplement the FFP classification in the sense of modifiers with additional edema detection. This aspect would require further research.

Whether the results of the MRI really only resulted in a change of the therapy regime in 10% (n = 7/67) cannot be answered retrospectively, since no documentation was available as to which operative planning was previously available.

In the case of a one-sided fracture of the sacrum, prophylactically stabilizing the opposite side with, for example, transiliosacral fixator internal, "sacral bar" or transiliosacral screw osteosynthesis remains controversial within the participating clinics. An evaluation of the therapy change from one-sided to bilateral stabilization of the sacrum is not possible in this study, but would be desirable for future studies.

For the clinical practice we recommend rapid MRI diagnostics for old patients without trauma or with inadequate trauma and pain in the pelvis, in order to correctly diagnose the extent of the fracture and to have prompt therapy without long immobilization times for the patient.

## Summary and conclusion

Study results suggest that MRI can detect a significantly higher number of FFPs as compared to conventional CT. In addition, fractures were classified into more severe injuries, which led to a more frequent surgical procedure. The evaluation of bone marrow changes by MRI or Dual Source CT is therefore recommended for early treatment decisions and operative planning.
